# Treadmill Exercise Enhances the Effects of Zoledronate on Bone Microarchitecture and Mechanical Strength in Ovariectomized Rat Model of Osteoporosis

**DOI:** 10.3390/jfmk11020159

**Published:** 2026-04-18

**Authors:** Yuta Tsubouchi, Takashi Kataoka, Ryota Takase, Takefumi Otsu, Ryoji Hamanaka, Masashi Kataoka, Nobuhiro Kaku

**Affiliations:** 1 Division of Human Biology, Department of Health Science, Oita University of Nursing and Health Sciences, 2944-9 Megusuno, Oita-City 870-1201, Oita, Japan; 2 Department of Orthopaedic Surgery, Faculty of Medicine, Oita University, 1-1 Idaigaoka, Hasama-machi, Yufu-City 879-5593, Oita, Japan; 3 Oita University Hospital Rehabilitation Center, 1-1 Idaigaoka, Hasama-machi, Yufu-City 879-5593, Oita, Japan; 4 Division of Mechatronics, Department of Innovative Engineering, Faculty of Science and Technology, Oita University, 700 Dannoharu, Oita-City 870-1192, Oita, Japan; 5 Physical Therapy Course of Study, Faculty of Welfare and Health Sciences, Oita University, 700 Dannoharu, Oita-City 870-1192, Oita, Japan

**Keywords:** osteoporosis, zoledronate, treadmill exercise, bone microarchitecture, mechanical strength, ovariectomized rats, trabecular bone, bone remodeling

## Abstract

**Background:** The combination of pharmacological therapy and exercise is frequently recommended for osteoporosis management; however, whether antiresorptive agents may interfere with exercise-induced bone adaptation remains unclear. This study aimed to investigate the independent and combined effects of zoledronate and treadmill exercise on bone microarchitecture and mechanical strength in an ovariectomized rat model. **Methods:** Twenty-four female Sprague Dawley rats underwent ovariectomy and were assigned to four groups: Control, zoledronate (ZA), treadmill exercise (T), and combined zoledronate and exercise (ZA + T). An additional sham-operated group was included. Zoledronate was administered as a single subcutaneous injection, and a 6-week treadmill exercise routine was implemented. Bone microarchitecture was assessed using micro-computed tomography, and a three-point bending test was employed for evaluation of mechanical properties. **Results:** The combined ZA + T group demonstrated significant improvements in trabecular bone parameters, including bone volume/tissue volume and trabecular number, compared with the Control group. Mechanical strength parameters, including maximum load and stiffness, were also significantly enhanced in the ZA + T group. Cortical bone parameters exhibited no significant changes. **Conclusions:** Treadmill exercise did not attenuate the effects of zoledronate, and may offer additive benefits in enhancing trabecular bone microarchitecture and mechanical strength. These findings suggest that exercise therapy can complement bisphosphonate treatment and contribute to optimizing therapeutic strategies for osteoporosis, supporting the potential utility of combined pharmacological and exercise-based interventions for improving bone health.

## 1. Introduction

Osteoporosis is characterized by increased bone fragility due to age-related loss of bone mass and bone microarchitecture deterioration, resulting in an elevated risk of fractures even after minor trauma [1]. Osteoporotic fractures in older adults are associated with a decline in activities of daily living, prolonged bedridden status, and worsened prognosis, thereby posing significant challenges for both healthcare and long-term care systems [2]. In Japan, the number of patients with osteoporosis aged ≥40 years has been reported to be approximately 6.4 and 10.7 million based on lumbar spine and proximal femur measurements, respectively [3]. With the rapid progression of a super-aging society, the prevalence of osteoporosis and the incidence of related fractures are anticipated to further increase. These fractures are associated with high recurrence and mortality rates, imposing a considerable socioeconomic burden through increased medical and long-term care costs [4,5]. Osteoporosis has been recently recognized not merely as a condition of reduced bone mass but as a complex disorder involving bone quality deterioration. As understanding of bone microarchitecture and bone remodeling dynamics advances, the significance of nonpharmacological interventions, including exercise therapy, in addition to pharmacological treatment has garnered increasing attention. Therefore, developing effective therapeutic strategies for maintaining bone metabolism and preventing fractures has become an urgent issue.

Pharmacological therapy remains the cornerstone of osteoporosis treatment, among which bisphosphonates (BPs) are broadly used as first-line agents with well-established fracture prevention efficacy. BPs demonstrate a high affinity for hydroxyapatite and accumulate on bone surfaces. During the process of bone resorption, they are taken up by osteoclasts, where they inhibit the mevalonate pathway, particularly the activity of farnesyl pyrophosphate synthase, thereby inducing osteoclast dysfunction and apoptosis [6]. Through this mechanism, BPs suppress bone resorption and decrease bone turnover. Evidence from animal studies has indicated that zoledronate administration in ovariectomized rat models preserves trabecular bone mass and microarchitecture and prevents reductions in the mechanical strength of the tibia and femur [7]. These findings suggest that BPs can contribute to maintaining bone mass and preserving bone quality. Similarly, clinical studies have revealed that BP agents, including alendronate, risedronate, and zoledronate, significantly mitigate the risk of both vertebral and nonvertebral fractures, and their anti-fracture efficacy is believed to contribute to improved long-term outcomes in patients with osteoporosis [8,9,10]. Although rare but life-threatening adverse events, including BP-related osteonecrosis of the jaw and atypical femoral fractures, have been reported with long-term use, BPs continue to be broadly prescribed worldwide owing to their favorable risk–benefit profile in osteoporosis management [11].

In addition to pharmacological treatment, exercise therapy plays a crucial role in osteoporosis management. Although pharmacological interventions are frequently initiated following the occurrence of fragility fractures, exercise therapy can be implemented at earlier stages of bone loss or as a preventive strategy before fractures occur [12,13]. Exercise contributes to improvements in bone strength and offers added benefits, including fall prevention and physical function maintenance. Evidence from animal studies has demonstrated that treadmill exercise in ovariectomized rats significantly improves trabecular microarchitecture in the proximal femur and increases mechanical strength, including maximal load capacity. Similarly, in experimental models, jump exercise loading has been shown to stimulate bone formation [14,15]. Furthermore, clinical studies have supported the beneficial effects of exercise therapy. In postmenopausal females (mean age, 65.8 years), exercise has been reported to heighten trabecular bone mineral density (BMD) at the distal tibia and cortical BMD at the tibial shaft, as measured using peripheral quantitative computed tomography (CT), by 0.87% and 0.89%, respectively [16]. The main mechanism underlying these osteogenic effects is believed to involve the suppression of sclerostin expression derived from osteocytes. Mechanical loading activates the Wnt/β-catenin signaling pathway, which promotes bone modeling and ultimately improves bone microarchitecture and mechanical strength [17].

In clinical practice, BP therapy combined with exercise is frequently recommended to maximize fracture prevention in patients with osteoporosis. However, the antiresorptive effects of BPs and the mechanical loading-induced osteogenic effects may involve distinct and potentially opposing mechanisms of bone remodeling. Previous studies have mainly focused on bone mass or bone mineral content (BMC), and comprehensive evaluations of trabecular microarchitecture, cortical bone, and mechanical strength under combined therapy remain limited [18,19]. Therefore, whether the suppression of bone resorption by BPs could attenuate the adaptive bone-forming responses induced by exercise remains unclear.

Accordingly, this study aimed to investigate the independent and combined effects of zoledronate administration and treadmill exercise using an ovariectomized rat model. Bone microarchitecture was assessed using micro-CT analysis, and mechanical properties were evaluated using biomechanical testing. Through these analyses, we sought to determine whether combined pharmacological and mechanical interventions could improve bone morphological and mechanical properties. However, the independent and combined effects of zoledronate and exercise on bone microarchitecture and mechanical strength have not been fully elucidated, particularly using a factorial experimental design. We hypothesized that combined zoledronate administration and treadmill exercise would result in greater improvements in bone microarchitecture and mechanical strength than either intervention alone, without negatively interfering with each other’s effects.

## 2. Materials and Methods

### 2.1. Surgical Techniques Used for Ovariectomy

The Oita University Institutional Animal Ethics Committee approved all animal experiments (approval number: 1624001). Ovariectomy was performed as previously described [20]. Rats were anesthetized by intraperitoneal injection of a mixed anesthetic comprising medetomidine (0.15 mg/kg), midazolam (2 mg/kg), and butorphanol (2.5 mg/kg) at total volumes of 0.3–0.4 mL. All surgical procedures were performed under standard sterile conditions. The rats were placed in the supine position, and the abdominal area was disinfected. A midline abdominal skin incision (approximately 2 cm long) was created using a scalpel. To expose the right subperitoneal ovary, the peritoneum was carefully incised and gently spread using forceps. The surrounding adipose tissue was grasped using atraumatic forceps and gently exteriorized to facilitate the elevation of the ovary, oviduct, and a portion of the uterus. The oviduct and the associated blood vessels beneath the ovary were ligated using silk sutures, and the ovaries were excised. Following ovariectomy, the proximal end of the oviduct was returned to the peritoneal cavity. Subsequently, the left ovary was excised using the same procedure. The peritoneum and skin were closed using 3-0 nylon sutures.

In the sham group, the ovaries were exteriorized similarly but were returned to the peritoneal cavity without excision. The peritoneum and skin were closed using identical suturing techniques. Ovariectomized and sham rats were maintained postoperatively for 8 weeks; during this period, body weight was measured to confirm postoperative weight gain.

### 2.2. Experimental Design

**Figure 1:**
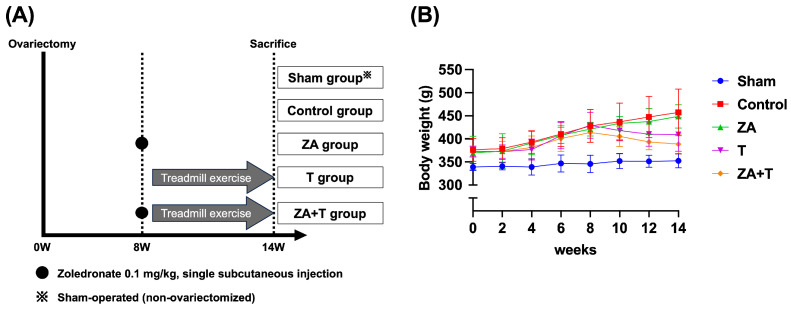
Experimental design and changes in body weight. (**A**) Schematic overview of the experimental protocol. All rats underwent bilateral ovariectomy at week 0 to induce osteoporosis. At 8 weeks post-ovariectomy, rats were randomly assigned to four intervention groups (*n* = 5 per group): Control group (saline administration), zoledronate group (ZA; single subcutaneous injection of zoledronate, 0.1 mg/kg), treadmill exercise group (T), and zoledronate combined with treadmill exercise group (ZA + T). Treadmill exercise was performed for 6 weeks, including a 1-week acclimation period (60 min/day, 5 days/week, maximum speed of 20 m/min). Zoledronate was administered as a single subcutaneous injection at week 8. All rats were euthanized at week 14 post-ovariectomy. An additional sham-operated group without ovariectomy (Sham group) was included. A formal a priori sample-size calculation was not performed. The sample size was determined on the basis of previous animal studies using similar osteoporosis models and following ethical considerations to minimize the number of animals used. (**B**) Longitudinal changes in body weight in each group throughout the experimental period. Body weight was measured weekly to confirm the validity of the ovariectomy model.

This study used 24 female Sprague Dawley rats aged 24 weeks. All animals underwent bilateral ovariectomy to induce osteoporosis. At 8 weeks post-ovariectomy, the rats were randomly assigned to four intervention groups (*n* = 5 per group) using simple randomization with a computer-generated random number sequence by an investigator not involved in subsequent analyses: Control (Control group; saline administration), zoledronate (ZA group; zoledronate 0.1 mg/kg, single subcutaneous injection; Novartis Pharma K.K., Tokyo, Japan), treadmill exercise (T group), and zoledronate administration combined with treadmill exercise (ZA + T group) (Figure 1A). Moreover, four age-matched rats without ovariectomy were included as a sham-operated group (Sham group).

Zoledronate was administered as a single subcutaneous injection to the ZA and ZA + T groups at 8 weeks post-ovariectomy. Zoledronate dose and administration regimen were determined on the basis of our previous studies [21]. At the same time point, treadmill exercise was initiated in the T and ZA + T groups. Before the intervention period, the rats in the T and ZA + T groups underwent a 1-week acclimation period on a rodent treadmill (MK-680; Muromachi Kikai, Tokyo, Japan). During acclimation, the animals ran for 10–15 min/day at a 5–10 m/min speed with a 0° incline. Following the acclimation period, treadmill exercise was continued for an additional 5 weeks, resulting in a 6-week total exercise period. The exercise protocol comprised running at a maximum speed of 20 m/min on a 0° incline for 60 min/days, 5 days/week. The exercise protocol was determined on the basis of previously published studies using treadmill exercise in rat models [18].

Body weight was measured weekly throughout the experimental period to monitor postoperative changes and to confirm the validity of the ovariectomy model. The longitudinal changes in body weight are presented in Figure 1B.

All animals were euthanized at 14 weeks post-ovariectomy (aged 38 weeks), corresponding to 6 weeks following the initiation of zoledronate administration and treadmill exercise. Euthanasia was performed under deep anesthesia using the same anesthetic protocol as that used for the ovariectomy procedure. Blood samples were collected by cardiac puncture under anesthesia, and the right femur and gastrocnemius muscle were harvested for subsequent analyses.

### 2.3. High-Resolution Micro-CT Analysis

Bone micro-CT imaging was performed according to established guidelines [22]. The explanted specimens were scanned using a SkyScan 1172 (Bruker, Kontich, Belgium) with a 20 µm voxel size. Data acquisition was conducted at 100 kV and 100 mA, and the images were reconstructed using a cone-beam algorithm.

Each specimen was positioned on the object stage, and scanning was performed over a 180° rotation with a 105 ms exposure time. Data analysis was performed using a CT Analyzer (Bruker, Kontich, Belgium). The region of interest for femur bone morphometry was defined as 0.8–3.8 mm proximal to the growth-cartilage plate for trabecular bone analysis and 3.0–3.8 mm for cortical bone analysis, and the tissue area in each slice was manually specified. The following parameters were measured: tissue volume (TV), bone volume (BV), BV fraction (BV/TV), trabecular bone thickness (Tb.Th), trabecular bone number (Tb.N), trabecular bone separation (Tb.Sp), cortical bone area (Cr.Ar), and cortical bone thickness (Cr.Th).

Cortical and trabecular BMDs were calculated using calibration with a reference phantom containing hydroxyapatite embedded in water-equivalent plastic, which was scanned together with the samples. The CT values obtained from the phantom were used for converting the measured CT values of the bone samples into BMD.

### 2.4. Biomechanical Analysis

The biomechanical properties of the femur were assessed using a three-point bending test with a universal material testing system (Instron 5865; Instron, Kanagawa, Japan). Before mechanical testing, the longitudinal and transverse diameters of the mid-diaphysis of each femur were measured using digital calipers.

For the three-point bending test, each femur was positioned on the sample supports with the anterior surface facing upward and the midpoint of the femur shaft aligned with the center of the supports. A load was applied at a constant displacement rate of 1 mm/s until a fracture occurred. The yield load was determined from the load–displacement curve that was obtained in the elastic deformation region.

Mechanical parameters, including stiffness and Young’s modulus, were calculated using the load–displacement data and from the previously measured longitudinal and transverse diameters. All biomechanical parameters were derived using the Instron software (Bluehill, Instron, Kanagawa, Japan).

### 2.5. Body Weight and Gastrocnemius Muscle Measurement

Body weight was measured for each rat immediately before euthanasia. The gastrocnemius muscle was carefully excised from the surrounding tissues, and connective tissues were removed. The gastrocnemius muscle wet weight was subsequently measured. The relative gastrocnemius muscle weight was calculated by normalizing the muscle wet weight to the body weight to account for inter-individual differences in body size.

### 2.6. Serum Marker Level Measurement

Serum samples collected by cardiac puncture during euthanasia were used for biomarker analyses. Blood samples were centrifuged to obtain serum, which was stored at −80 °C until analysis. A commercial laboratory (SRL Inc., Tokyo, Japan) measured the serum adiponectin and type I collagen C-terminal telopeptide (1CTP) levels. Adiponectin and 1CTP levels were measured using latex immunoturbidimetry and the RIA-2 antibody method, respectively. Measurements were performed in three rats per group owing to a limited serum volume available for outsourced laboratory analysis.

### 2.7. Statistical Analysis

All animals were included in the analyses, and no animals or samples were excluded from this study. Statistical analyses were performed using GraphPad Prism (version 11.0; GraphPad Software, San Diego, CA, USA). Group comparisons, including sham-operated animals, were performed for micro-CT-derived bone morphometric parameters, biomechanical testing outcomes, body weight, and gastrocnemius muscle wet weight. Serum biomarkers were measured using enzyme-linked immunosorbent assay (ELISA). Owing to the limited serum volume, ELISA measurements were performed in three rats per group (*n* = 3).

Data are presented as means ± standard deviations. Differences among groups were initially analyzed using one-way analysis of variance (ANOVA), followed by the Tukey–Kramer post hoc test for multiple comparisons when appropriate.

To further evaluate the independent and combined effects of zoledronate and exercise, a two-way ANOVA was additionally performed using the four ovariectomized (OVX) groups (Control, ZA, T, and ZA + T), with zoledronate treatment (yes/no) and exercise (yes/no) as factors. The main effects of each factor and their interaction were assessed. A *p*-value of <0.05 was considered statistically significant.

As an a priori sample size calculation was not performed, a post hoc power analysis was conducted using the G*Power software (version 3.1). BV/TV obtained from micro-CT analysis was defined as the primary outcome measure. Based on the observed effect size (f = 1.17), the statistical power for detecting differences among groups was 0.99 at an alpha level of 0.05, indicating sufficient statistical power for the primary outcome.

## 3. Results

### 3.1. BMD

**Figure 2:**
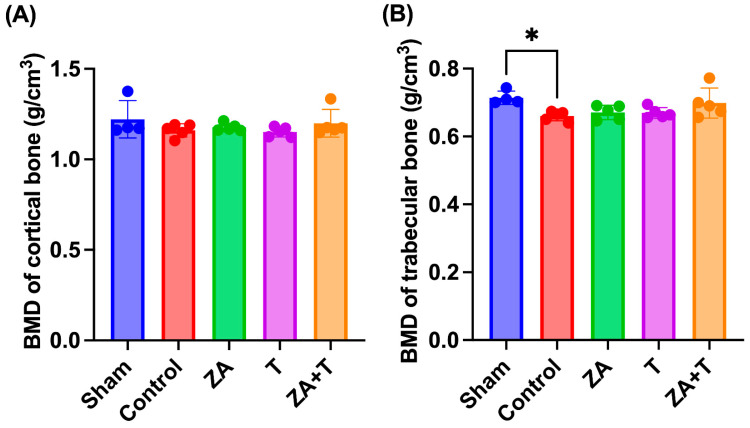
Bone mineral density (BMD) measured using micro-CT. (**A**) Cortical BMD and (**B**) trabecular BMD. BMD is expressed in g/cm^3^ as provided by the micro-CT analysis software (CTAn, version 1.15, Bruker, Kontich, Belgium). Data are presented as means ± standard deviations (SDs). Statistical analysis was performed using one-way analysis of variance (ANOVA), followed by the Tukey–Kramer post hoc test. * *p* < 0.05. *n* = 4 for the Sham group; *n* = 5 for the other groups.

The cortical and trabecular BMDs measured using micro-CT are depicted in Figure 2. No significant differences in cortical BMD were observed among the groups (Figure 2A). By contrast, the Control group exhibited a significantly lower trabecular BMD than the Sham group (*p* < 0.05). However, no significant differences were observed among the Control, ZA, T, and ZA + T groups (Figure 2B).

**Figure 3:**
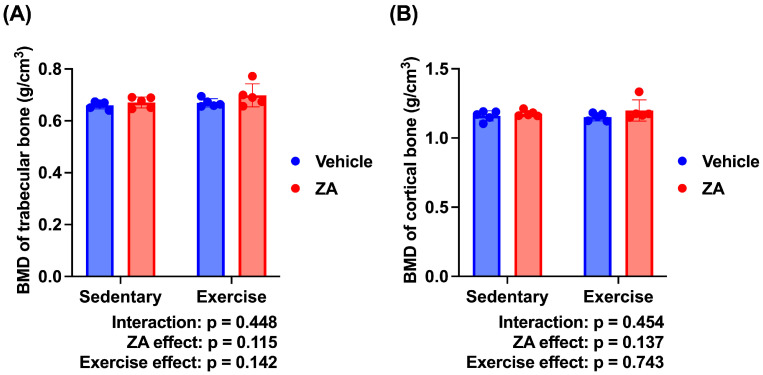
Bone mineral density (BMD) analyzed using two-way analysis of variance (ANOVA) in ovariectomized (OVX) rats. (**A**) Trabecular BMD and (**B**) cortical BMD. Data are presented as means ± standard deviations (SDs). Statistical analysis was performed using two-way ANOVA with zoledronate treatment (ZA) and exercise as factors. The main effects of ZA and exercise, as well as their interaction, are indicated in each panel. *n* = 5 per group.

To further evaluate the independent and combined effects of zoledronate and exercise, a two-way ANOVA was performed using the four OVX groups. For trabecular BMD, no significant main effects of zoledronate or exercise were observed, and no significant interaction effect was detected (Figure 3A). Similarly, for cortical BMD, neither the main effects of zoledronate nor exercise were significant, and no interaction effect was found (Figure 3B).

### 3.2. Trabecular Bone Microarchitecture

**Figure 4:**
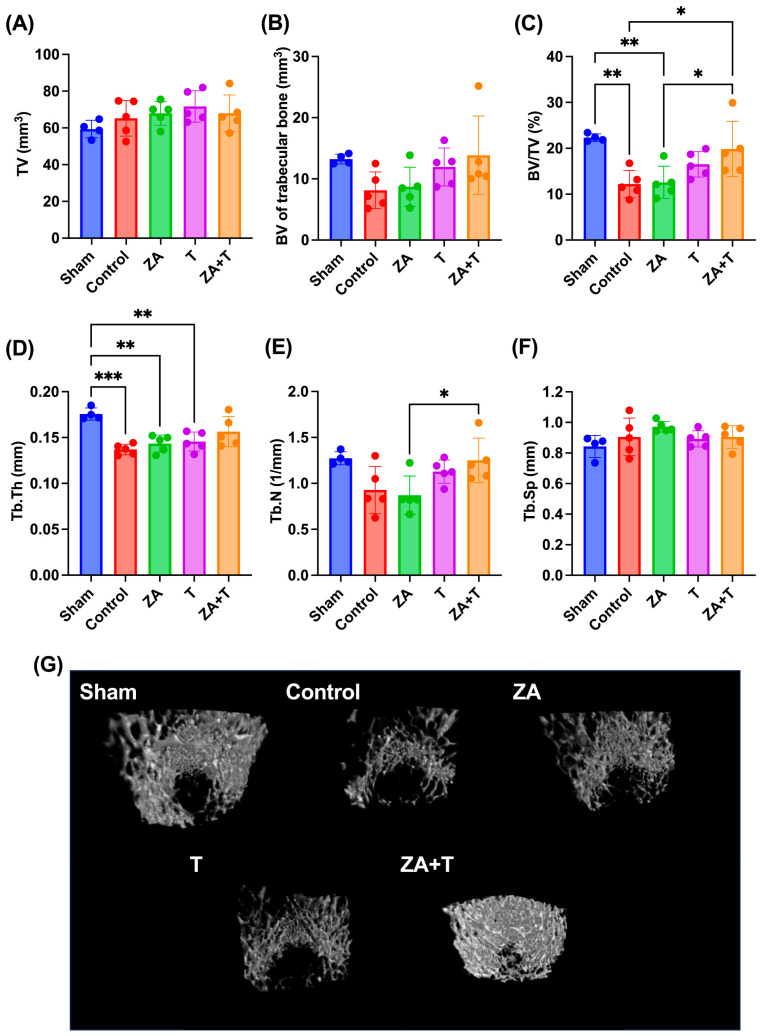
Trabecular bone microarchitecture assessed using micro-CT. (**A**) Total volume (TV), (**B**) bone volume (BV), (**C**) bone volume fraction (BV/TV), (**D**) trabecular bone thickness (Tb.Th), (**E**) trabecular bone number (Tb.N), and (**F**) trabecular bone separation (Tb.Sp). (**G**) Representative three-dimensional micro-CT images of the trabecular bone. Data are presented as means ± SDs. Statistical analysis was performed using one-way ANOVA, followed by the Tukey–Kramer post hoc test. * *p* < 0.05, ** *p* < 0.01, *** *p* < 0.001. *n* = 4 for the Sham group; *n* = 5 for the other groups.

The micro-CT-derived trabecular bone morphometric parameters are presented in Figure 4. The Control and ZA groups demonstrated a significantly lower BV/TV than the Sham group (*p* < 0.01). By contrast, the ZA + T group showed a significantly higher BV/TV than the Control and ZA groups (*p* < 0.05) (Figure 4C). The Control (*p* < 0.001), ZA (*p* < 0.01), and T groups (*p* < 0.01) showed a significantly lower Tb.Th than the Sham group (Figure 4D). The ZA + T group exhibited a significantly higher Tb.N than the ZA group (*p* < 0.05) (Figure 4E). No significant differences were observed among the groups in terms of TV, BV, and Tb.Sp (Figure 4A,B,F). The representative three-dimensional micro-CT images of trabecular bone are shown in Figure 4G.

**Figure 5:**
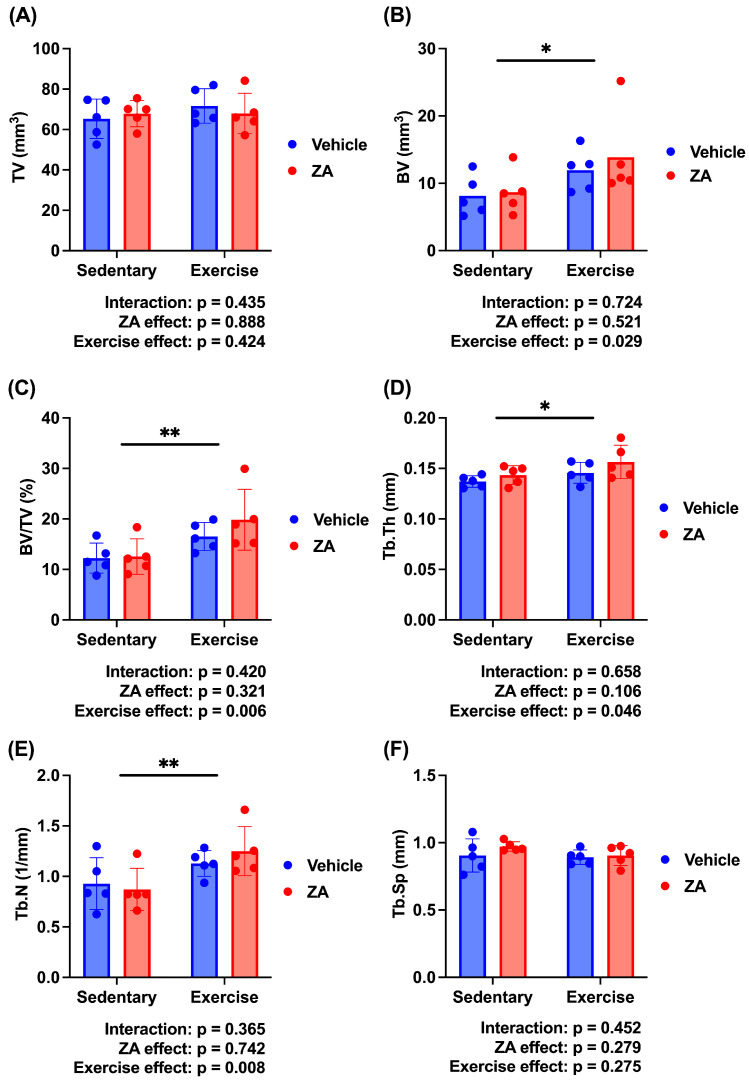
Trabecular bone microarchitecture analyzed using two-way analysis of variance (ANOVA) in ovariectomized (OVX) rats. (**A**) Tissue volume (TV), (**B**) bone volume (BV), (**C**) bone volume fraction (BV/TV), (**D**) trabecular thickness (Tb.Th), (**E**) trabecular number (Tb.N), and (**F**) trabecular separation (Tb.Sp). Data are presented as means ± standard deviations (SDs). Statistical analysis was performed using two-way ANOVA with zoledronate treatment (ZA) and exercise as factors. Significant main effects are indicated in each panel. * *p* < 0.05, ** *p* < 0.01. *n* = 5 per group.

To further evaluate the independent and combined effects of zoledronate and exercise, a two-way ANOVA was performed using the four OVX groups (Control, ZA, T, and ZA + T). Significant main effects of exercise were observed for BV (*p* < 0.05), BV/TV (*p* < 0.01), Tb.Th (*p* < 0.05), and Tb.N (*p* < 0.01), whereas no significant main effects of zoledronate or interaction effects were detected (Figure 5B–E). No significant main or interaction effects were observed for TV or Tb.Sp (Figure 5A,F).

### 3.3. Cortical Bone Morphometry

**Figure 6:**
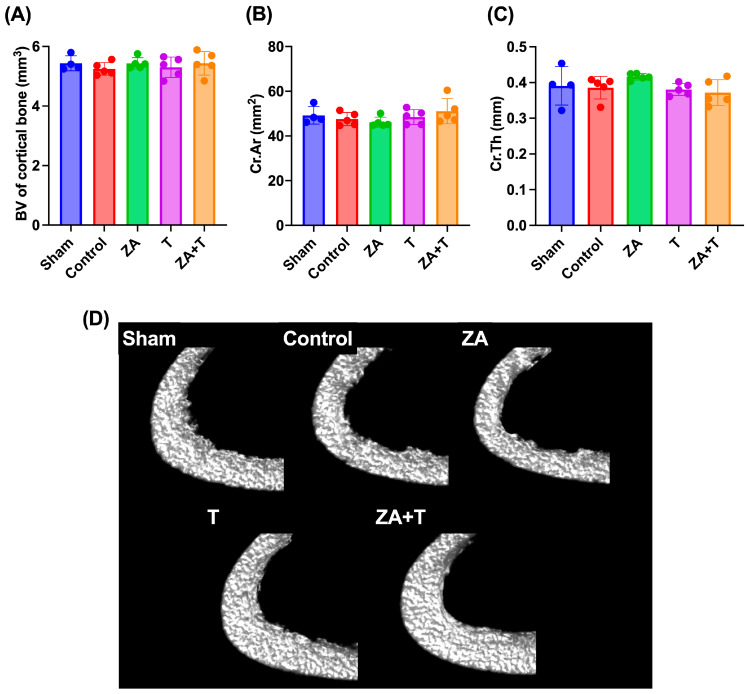
Cortical bone morphometric parameters assessed using micro-CT. (**A**) Cortical BV, (**B**) cortical bone area (Cr.Ar), and (**C**) cortical bone thickness (Cr.Th). (**D**) Representative cross-sectional micro-CT images of the cortical bone. Data are presented as means ± SDs. *n* = 4 for the Sham group; *n* = 5 for the other groups.

The cortical bone morphometric parameters are depicted in Figure 6. No significant differences were observed among the groups in terms of cortical BV, Cr.Ar, and Cr.Th (Figure 6A–C). Representative cross-sectional micro-CT images of cortical bone are presented in Figure 6D.

**Figure 7:**
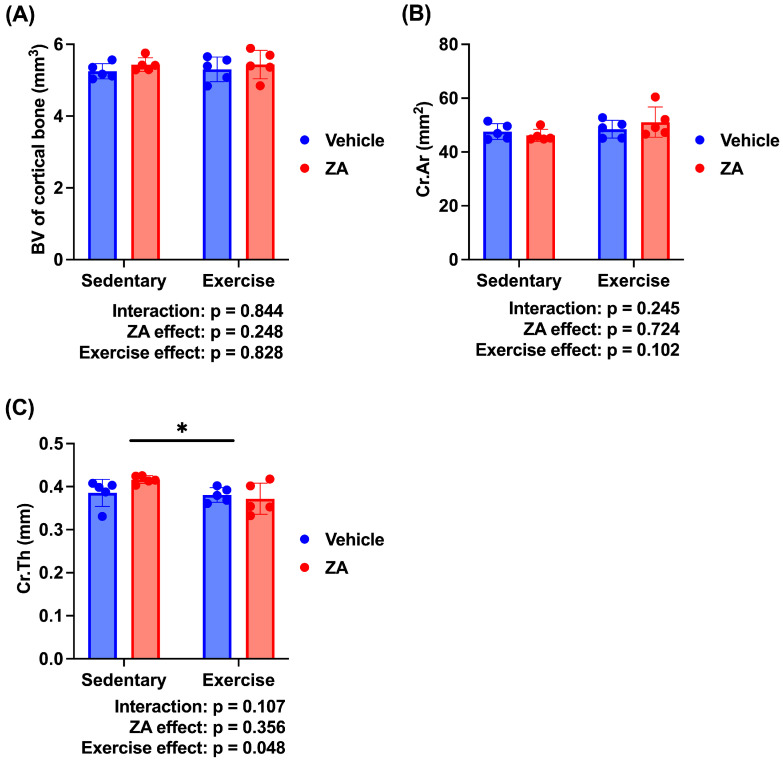
Cortical bone morphometric parameters analyzed using two-way analysis of variance (ANOVA) in ovariectomized (OVX) rats. (**A**) Cortical bone volume (BV), (**B**) cortical area (Cr.Ar), and (**C**) cortical thickness (Cr.Th). Data are presented as means ± standard deviations (SDs). Statistical analysis was performed using two-way ANOVA with zoledronate treatment (ZA) and exercise as factors. Significant main effects are indicated in each panel. * *p* < 0.05. *n* = 5 per group.

To further evaluate the independent and combined effects of zoledronate and exercise, a two-way ANOVA was performed using the four OVX groups. A significant main effect of exercise was observed for Cr.Th (*p* < 0.05), whereas no significant main effects of zoledronate or interaction effects were detected (Figure 7C). No significant main or interaction effects were observed for cortical BV or Cr.Ar (Figure 7A,B).

### 3.4. Mechanical Strength

**Figure 8:**
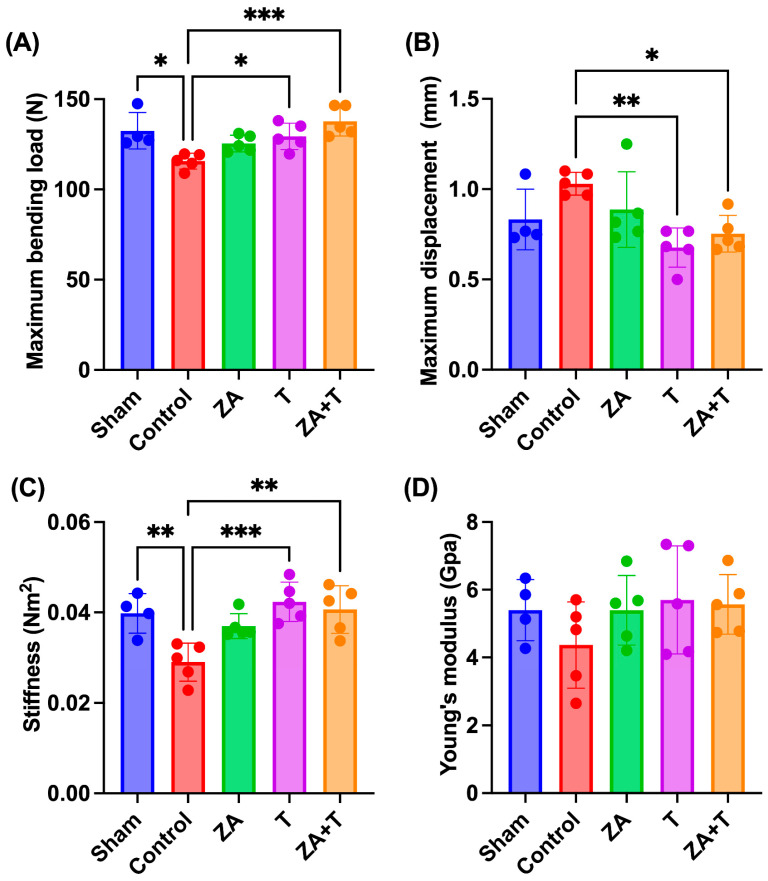
Mechanical properties of the femur assessed using the three-point bending test. (**A**) Maximum bending load, (**B**) maximum displacement, (**C**) stiffness, and (**D**) Young’s modulus. Data are presented as means ± SDs. Statistical significance was determined using one-way ANOVA, followed by the Tukey–Kramer post hoc test. * *p* < 0.05, ** *p* < 0.01, *** *p* < 0.001. *n* = 4 for the Sham group; *n* = 5 for the other groups.

The three-point bending test results are shown in Figure 8. The Sham (*p* < 0.05), T (*p* < 0.05), and ZA + T groups (*p* < 0.001) demonstrated a significantly higher maximum bending load than the Control group (Figure 8A). The T (*p* < 0.01) and ZA + T groups (*p* < 0.05) exhibited a significantly lower maximum displacement than the Control group (Figure 8B). The Sham (*p* < 0.01), T (*p* < 0.001), and ZA + T groups (*p* < 0.01) had a significantly higher stiffness than the Control group (Figure 8C). No significant differences in Young’s modulus were noted among the groups (Figure 8D).

**Figure 9:**
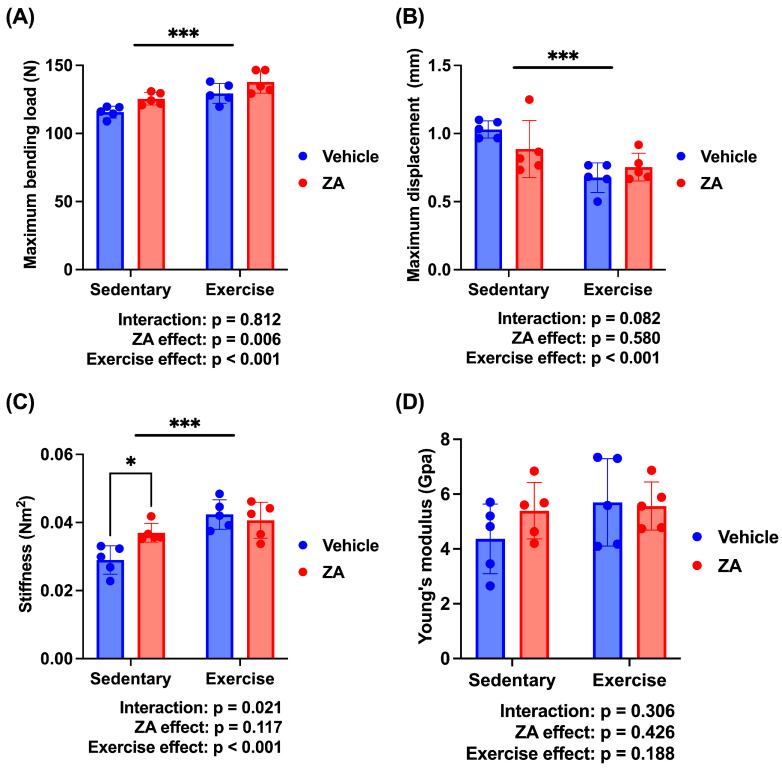
Biomechanical properties analyzed using two-way analysis of variance (ANOVA) in ovariectomized (OVX) rats. (**A**) Maximum bending load, (**B**) maximum displacement, (**C**) stiffness, and (**D**) Young’s modulus. Data are presented as means ± standard deviations (SDs). Statistical analysis was performed using two-way ANOVA with zoledronate treatment (ZA) and exercise as factors. Significant main and interaction effects are indicated in each panel. * *p* < 0.05, *** *p* < 0.001. *n* = 5 per group.

To further evaluate the independent and combined effects of zoledronate and exercise, a two-way ANOVA was performed using the four OVX groups. Significant main effects of exercise were observed for maximum bending load (*p* < 0.001), maximum displacement (*p* < 0.001), and stiffness (*p* < 0.001) (Figure 9A–C). A significant main effect of zoledronate was observed for maximum bending load (*p* < 0.01). In addition, a significant interaction effect was detected for stiffness (*p* < 0.05). No significant main or interaction effects were observed for Young’s modulus (Figure 9D).

### 3.5. Body Weight and Gastrocnemius Muscle Measurements

**Figure 10:**
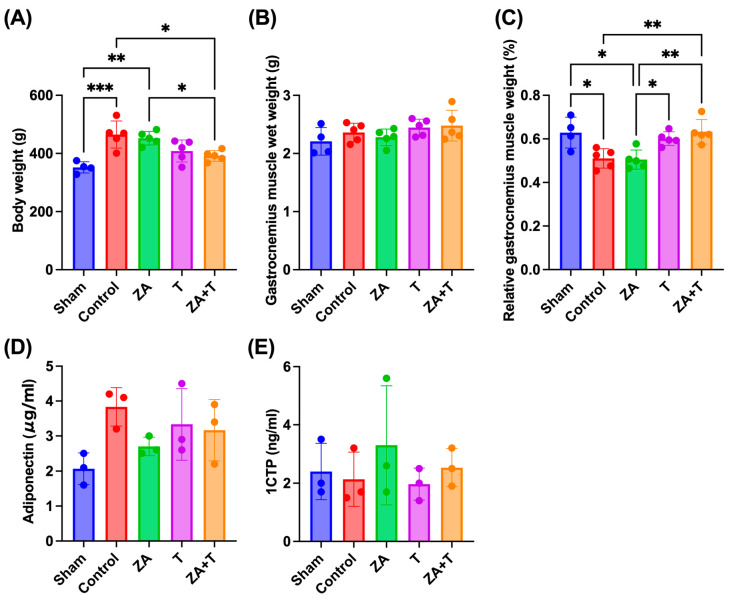
Body weight, gastrocnemius muscle measurements, and serum biomarkers. (**A**) Body weight, (**B**) gastrocnemius muscle wet weight, and (**C**) relative gastrocnemius muscle weight normalized to body weight are shown. (**D**) Serum adiponectin and (**E**) type I collagen C-terminal telopeptide levels measured using enzyme-linked immunosorbent assay (ELISA) are also presented. Data are presented as means ± SDs. Statistical analysis was performed using one-way ANOVA, followed by the Tukey–Kramer post hoc test. * *p* < 0.05, ** *p* < 0.01, *** *p* < 0.001. *n* = 4 for the Sham group; *n* = 5 for the other groups. ELISA measurements were performed in three rats per group (*n* = 3).

The body weight, gastrocnemius muscle wet weight, and relative gastrocnemius muscle weight are shown in Figure 10A–C. The Control (*p* < 0.001) and ZA groups (*p* < 0.01) showed a significantly higher body weight than the Sham group. Moreover, the Control (*p* < 0.05) and ZA groups (*p* < 0.05) exhibited a significantly higher body weight than the ZA + T group (Figure 10A).

No significant differences were observed in gastrocnemius muscle wet weight among the groups (Figure 10B). The Control (*p* < 0.05) and ZA groups (*p* < 0.05) demonstrated a significantly lower relative gastrocnemius muscle weight than the Sham group. Furthermore, the ZA group showed a significantly higher relative gastrocnemius muscle weight than the T group (*p* < 0.05). Additionally, the Control and ZA groups exhibited significantly higher values than the ZA + T group (*p* < 0.01) (Figure 10C).

### 3.6. Serum Biomarkers

The serum adiponectin and 1CTP levels measured using ELISA are shown in Figure 10D,E. No significant differences in adiponectin or 1CTP levels were noted among the groups.

## 4. Discussion

This study investigated the independent and combined effects of zoledronate administration and treadmill exercise on bone microarchitecture and mechanical strength in an ovariectomized rat model of osteoporosis. The discussion is structured to first summarize the principal findings, followed by interpretation of trabecular and cortical bone responses, mechanical properties, and secondary outcomes.

The principal finding of this study was that treadmill exercise did not attenuate the antiresorptive effects of zoledronate and could instead offer additive benefits for bone structural and mechanical properties. In particular, compared with pharmacological treatment alone, zoledronate administration combined with treadmill exercise significantly improved trabecular bone microarchitecture and mechanical strength. These findings suggest that mechanical loading through exercise can complement pharmacological therapy without compromising the therapeutic effects of BPs. This interpretation is further supported by two-way ANOVA, which demonstrated significant main effects of exercise without significant interaction effects, indicating that the combined intervention produced additive rather than synergistic effects.

This study revealed that zoledronate administration combined with treadmill exercise yielded additive improvements in trabecular bone microarchitecture and mechanical strength compared with either intervention alone. This finding is noteworthy because previous studies investigating combined pharmacological and exercise interventions have predominantly focused on changes in BMC, whereas comprehensive analyses of trabecular microarchitectural parameters remain relatively scarce [18].

BPs accumulate on bone surfaces and are taken up by osteoclasts during bone resorption, where they inhibit the mevalonate pathway and induce osteoclast apoptosis. Through this mechanism, BPs suppress bone resorption and enhance bone structure and mechanical strength. BPs suppress bone resorption by inhibiting osteoclast activity through the mevalonate pathway [6], whereas exercise promotes bone formation via mechanical loading-induced suppression of sclerostin and activation of the Wnt/β-catenin signaling pathway [17,23,24]. These processes correspond to remodeling-based and modeling-based bone formation, respectively [25]. Because these interventions act through distinct mechanisms and at different skeletal sites, their combination may exert complementary effects on bone microarchitecture and mechanical strength [18]. Consequently, their combination may exert additive effects on trabecular microarchitecture and mechanical strength while preserving each other’s biological actions. Nevertheless, this study was restricted to morphological assessments using micro-CT and biomechanical testing. Serum bone metabolic markers and histological analyses were not comprehensively investigated. Therefore, the previously proposed mechanistic interpretations remain speculative. To clarify the biological interactions between BP therapy and exercise-induced mechanical loading, further studies incorporating molecular and histological analyses will be crucial. Accordingly, the present findings should be interpreted as preliminary experimental evidence suggesting the potential benefits of combined pharmacological and mechanical interventions for improving bone structural and mechanical properties.

In this study, zoledronate monotherapy did not generate significant improvements in bone microarchitecture or mechanical strength following ovariectomy. This finding contrasts with previous studies reporting marked bone-protective effects of zoledronate in similar animal models. For example, using in vivo micro-CT, Brouwers et al. demonstrated that early administration of zoledronate at 2 weeks following ovariectomy restored trabecular microarchitecture to near baseline levels, whereas delayed administration at 8 weeks caused only partial recovery [26]. Differences in dosing regimen, administration timing, and observation duration may explain these discrepancies. Furthermore, the relatively low dose and single-administration protocol used in the present study may have contributed to the limited response. In this study, zoledronate was administered at a relatively late stage following ovariectomy, and the observation period was limited to 6 weeks. Therefore, the limited efficacy observed in the ZA group may reflect insufficient time for the full pharmacological effects to manifest. To elucidate the time-dependent effects of zoledronate on bone structural and mechanical properties, longer-term studies are warranted.

Notably, the improvements in trabecular bone microarchitecture observed in this study were accompanied by enhanced mechanical strength, suggesting that the combined intervention of zoledronate administration and treadmill exercise translated into functionally meaningful skeletal benefits. Consistent with these observations, two-way ANOVA revealed significant main effects of exercise on trabecular parameters, whereas no interaction effects between zoledronate and exercise were detected. This suggests that exercise contributed independently to the observed improvements in bone microarchitecture. In contrast, no significant changes were observed in Young’s modulus, suggesting that the intrinsic material properties of bone were not altered by the interventions. These findings indicate that the observed improvements in mechanical strength may primarily reflect changes in bone structural organization, rather than alterations in the material properties of bone tissue. The lack of significant changes in BMD despite improvements in trabecular microarchitecture may be explained by differences in the nature of these measurements. BMD derived from micro-CT primarily reflects mineral density based on X-ray attenuation values, whereas parameters such as BV/TV and trabecular number represent structural characteristics of bone. Therefore, improvements in bone microarchitecture may not necessarily be captured by densitometric measurements alone.

Remarkably, these beneficial effects were predominantly observed in the trabecular compartment, whereas cortical bone parameters were not significantly altered. This preferential response of the trabecular bone may be attributed to its higher metabolic activity and greater mechanosensitivity than the cortical bone [27,28]. The trabecular bone undergoes more rapid remodeling and is therefore more responsive to pharmacological and mechanical stimuli. By contrast, the cortical bone exhibits a slower turnover rate and may necessitate a longer intervention period or higher mechanical loading to demonstrate detectable structural changes. Moreover, the loading intensity applied in this study could be inadequate to induce periosteal adaptation in the cortical bone. Despite the limited effects observed in the cortical bone, the improvements in trabecular microarchitecture may have substantially contributed to the observed increases in mechanical strength. These findings align with previous studies indicating that mechanical loading-based interventions preferentially influence the trabecular bone, particularly under conditions of estrogen deficiency [19,29].

In this study, gastrocnemius muscle wet weight and serum biomarkers were evaluated as secondary outcomes. Although no significant differences were observed in absolute muscle wet weight, the combined intervention group tended to exhibit a higher relative muscle weight than the single-treatment groups. However, body composition and muscle function were not directly assessed; therefore, these findings should be interpreted with caution. The effects of bisphosphonates on skeletal muscle remain controversial. In vitro studies have suggested that alendronate can impair cytoskeletal organization and inhibit differentiation in undifferentiated human skeletal muscle cells, while exerting minimal effects on mature myotubes [30]. In contrast, clinical studies have reported associations between bisphosphonate therapy and increased skeletal muscle mass indices, potentially through indirect mechanisms such as improved bone metabolism and physical activity levels [31]. In the present study, the observed tendency toward increased relative muscle weight in the combined group may be considered hypothesis-generating, and the underlying mechanisms remain unclear. Although muscle–bone crosstalk mediated by myokines such as insulin-like growth factor 1 and myostatin has been proposed [32,33,34], further studies incorporating direct assessments of muscle mass and function are required to clarify these relationships.

Furthermore, serum adiponectin and 1CTP levels were assessed in a limited number of samples (*n* = 3 per group) as exploratory outcomes, in order to investigate potential changes in lipid metabolism and bone turnover associated with ovariectomy and exercise intervention. However, the groups demonstrated no significant differences, and the small sample size limited the interpretability of these findings. This lack of significant changes may be attributed to the relatively short intervention period and the low sensitivity of these systemic markers to detect localized changes in bone remodeling. These analyses were exploratory in nature and were not intended as primary outcome measures. Therefore, the skeletal muscle- and serum biomarker-related findings should be considered hypothesis-generating. To elucidate the interactions among muscles, bones, and adipose tissues, further studies with larger sample sizes and comprehensive analyses, including myokines and adipokines, are warranted.

The findings of this study have potential clinical implications for managing osteoporosis. The present results suggest that pharmacological therapy combined with mechanical loading can more effectively improve bone structural integrity and mechanical strength than either intervention alone, without compromising the antiresorptive effects of BPs. These findings support the clinical recommendation that exercise therapy should be encouraged, even during BP therapy, to optimize fracture prevention strategies.

However, this study has several limitations. First, a formal a priori sample size calculation was not performed, and the sample size was determined based on previous studies and ethical considerations to minimize animal use. Although post hoc power analysis indicated sufficient statistical power for the primary outcome, the possibility of type II errors for secondary outcomes cannot be excluded. Second, serum biomarker analyses were conducted in a limited number of animals (*n* = 3 per group) owing to insufficient sample volume, which may have reduced the sensitivity to detect significant differences in bone metabolism. Third, body composition and muscle function were not directly assessed; therefore, the interpretation of relative muscle weight should be made with caution. Fourth, although body weight changes were monitored to confirm the validity of the ovariectomy model, additional metabolic parameters were not evaluated. Fifth, mechanistic insights were limited, as molecular and histological analyses were not performed, and the lack of bone formation markers may have constrained the interpretation of bone remodeling dynamics. Sixth, the intervention period was relatively short, and longer-term effects of combined therapy on bone remodeling remain unclear. Finally, this was an experimental study using an ovariectomized rat model, and extrapolation of the findings to human clinical populations should be undertaken with caution. Despite these limitations, the present findings provide evidence supporting the potential benefits of combining bisphosphonate therapy with exercise. Further studies incorporating larger sample sizes, longer intervention periods, and comprehensive molecular, histological, and functional assessments are warranted to elucidate the underlying mechanisms and optimize therapeutic strategies for improving bone health.

## 5. Conclusions

This study demonstrated that zoledronate administration combined with treadmill exercise caused additive improvements in trabecular bone microarchitecture and mechanical strength in an ovariectomized rat model of osteoporosis. Notably, treadmill exercise did not attenuate the antiresorptive effects of zoledronate, suggesting that pharmacological and mechanical interventions can be effectively combined without compromising their respective benefits.

These findings suggest that exercise therapy can complement BP therapy, contributing to optimized bone quality and strength. Although the underlying mechanisms require further investigation, the present results offer preliminary evidence supporting the potential utility of combined therapeutic strategies for improving bone health and preventing fractures.

## Data Availability

The original contributions presented in this study are included in the article. Further inquiries can be directed to the corresponding authors.

## Abbreviations

The following abbreviations are used in this manuscript:

ADL	Activities of daily living
BMC	Bone mineral content
BMD	Bone mineral density
BP	Bisphosphonate
BV	Bone volume
BV/TV	Bone volume fraction
Cr.Ar	Cortical bone area
Cr.Th	Cortical bone thickness
ELISA	Enzyme-linked immunosorbent assay
FPPS	Farnesyl pyrophosphate synthase
IGF-1	Insulin-like growth factor 1
ROI	Region of interest
Tb.N	Trabecular bone number
Tb.Sp	Trabecular bone separation
Tb.Th	Trabecular bone thickness
TV	Tissue volume
1CTP	Type I collagen C-terminal telopeptide
